# Finite Element Analysis of Peri-Implant Stress in Maxillary All-on-Four Rehabilitation: Effects of Posterior Implant Angulation and Loading Protocol

**DOI:** 10.3390/ma19061239

**Published:** 2026-03-20

**Authors:** Juan Alberto Aristizábal-Hoyos, Leidy Katherine Gil-Tabares, Natalia Giraldo-Vélez, Martha Isabel Torres-Arteaga, Catalina Garces-Gonzalez, Olga Patricia López-Soto, Héctor Fuentes-Barría, Raúl Aguilera-Eguía, Lisse Angarita-Davila

**Affiliations:** 1 Departamento de Salud Oral, Facultad de Salud, Universidad Autónoma de Manizales, Manizales 170008, Colombia; jaristi@autonoma.edu.co (J.A.A.-H.); leidykatherinegil@hotmail.com (L.K.G.-T.); nata022@live.com (N.G.-V.); marthai.torresa@autonoma.edu.co (M.I.T.-A.); catalina.garcesg@autonoma.edu.co (C.G.-G.); sonrie@autonoma.edu.co (O.P.L.-S.); 2 Centro de Investigación en Medicina de Altura (CEIMA), Universidad Arturo Prat, Iquique 1110939, Chile; 3 Departamento de Salud Pública, Facultad de Medicina, Universidad Católica de la Santísima Concepción, Concepción 3349001, Chile; raguilerae@ucsc.cl; 4 Escuela de Nutrición y Dietética, Facultad de Medicina, Universidad Andres Bello, Concepción 3349001, Chile; lisse.angarita@unab.cl

**Keywords:** finite element analysis, jaw, edentulous, dental implants, immediate dental implant loading, rehabilitation

## Abstract

**Objective:** To evaluate the biomechanical effects of varying posterior implant inclinations and loading protocols on peri-implant stress distribution in full-arch maxillary rehabilitations using the All-on-Four concept. **Methodology:** A three-dimensional finite element model of an edentulous atrophic maxilla was developed from a digital point cloud. Four implants were placed according to the All-on-Four protocol: two anterior vertical implants and two posterior implants with inclinations of 0°, 15°, 30°, or 45°. Mini-abutments and a titanium bar prosthesis were included. Material properties were assumed as homogeneous, isotropic, and linearly elastic. Immediate loading was simulated using frictional contacts (µ = 0.3), whereas delayed loading assumed complete osseointegration (bonded contacts). The models were meshed using 10-node quadratic tetrahedral elements (SOLID187) in ANSYS^®^. Maximum von Mises stress in cortical bone, cancellous bone, implants, abutments, and the prosthetic bar was assessed. **Results:** Posterior implant tilt significantly reduced peri-implant stress. Under immediate loading, the highest stress occurred at 0° inclination in the posterior left implant (82.36 MPa) and decreased progressively with increasing tilt, reaching 33.63 MPa at 45° (≈59% reduction). Delayed loading generally produces lower stress magnitudes, particularly at extreme tilts. Anterior implants experienced lower stress levels across all configurations. Comparative analysis demonstrated that immediate loading increased stress at lower angulations, while differences between loading protocols were minimal at higher inclinations. **Conclusions:** Posterior implant angulation and loading protocol critically influence peri-implant stress distribution. Increased posterior tilt combined with appropriate loading reduces peak cortical bone stresses, supporting biomechanical optimization in All-on-Four maxillary rehabilitations.

## 1. Introduction

The rehabilitation of completely edentulous patients remains a major clinical challenge in implant dentistry due to anatomical constraints such as severe alveolar bone resorption, low bone quality, and pneumatization of the maxillary sinus [1]. Traditional surgical approaches, including sinus augmentation and bone grafting, are associated with increased morbidity, treatment time, and cost [2,3]. In contrast, prosthetic-driven implant strategies aim to maximize implant stability while minimizing surgical invasiveness.

The All-on-Four treatment concept, introduced by Maló and colleagues, represents a widely adopted strategy for full-arch fixed prosthetic rehabilitation in atrophic jaws. It consists of placing two vertically oriented anterior implants and two distally tilted posterior implants (up to 45°) with immediate loading of the prosthesis [4,5]. This configuration aims to increase anterior–posterior implant spread, reduce cantilever length, and leverage available cortical bone without extensive augmentation procedures [6,7]. Clinical reports demonstrate high implant survival rates and favorable functional outcomes with the All-on-Four technique in both maxillary and mandibular arches [8,9].

Despite its clinical success, biomechanical questions remain concerning the influence of implant angulation, loading protocol, and prosthetic design on stress distribution within peri-implant bone and prosthetic components. In particular, the degree of posterior implant tilt has been shown to affect the distribution of mechanical loads, with lower tilts often associated with higher peak stresses in peri-implant bone [10,11,12]. Several finite element analysis (FEA) studies have reported that posterior implants inclined at moderate angles (e.g., 30–45°) can reduce peri-implant stress concentrations relative to vertical implants, although the effects are sensitive to loading direction and prosthetic design [1,13].

Immediate loading protocols further complicate the biomechanical environment at the bone–implant interface. Early functional loading may increase micromotion at the implant interface, potentially affecting osseointegration [1,5]. Most biomechanical investigations of All-on-Four configurations under immediate loading have utilized FEA to predict stress distribution, revealing that peak stresses often occur around the tilted posterior implants and that implant inclination significantly affects stress magnitudes [1,5,13]. However, direct comparisons between immediate and delayed loading conditions in the same geometric model are relatively scarce in the literature [1].

Finite element analysis is an established computational tool for investigating internal stress and strain fields in dental implant systems, enabling the evaluation of multiple variables such as implant inclination, loading protocol, and material properties [1,5,13,14]. FEA allows researchers to simulate both idealized and clinically relevant conditions, providing insights that are difficult to capture in vitro or in vivo due to the complexity of biological tissues and ethical limitations [15].

In addition to implant inclination and loading time, other design factors such as implant–abutment connection type and framework geometry have also been investigated using FEA, indicating that prosthetic components influence stress transmission to the bone and implant interfaces (0, 2, 7). Recent predictive modeling studies further suggest that occlusal load direction may be as important as implant angulation in regulating stress distribution [16,17].

To address the gaps in current biomechanical evidence, this study used three-dimensional finite element analysis to evaluate the effects of varying posterior implant inclinations and loading protocols (immediate vs. delayed) on peri-implant stress distribution in an edentulous maxillary model following the All-on-Four concept. Through comparative analysis of these configurations, the present study aims to inform optimal implant angulation strategies and loading protocols to improve the biomechanical performance of full-arch fixed prostheses.

## 2. Materials and Methods

### 2.1. Three-Dimensional FEA Modeling

This computational experimental study developed a three-dimensional finite element model of a semi-complete skull focusing on the maxilla. The anatomical geometry was reconstructed from a point cloud obtained from Turbosquid^®^ (TurboSquid, New Orleand, LA, USA). The selected geometry was based on a standardized edentulous maxillary model and does not correspond to any identifiable patient; therefore, ethical approval was not required.

To ensure anatomical plausibility, the reconstructed geometry was dimensionally verified against anatomical measurements reported in the literature. Surface reconstruction and Boolean operations are commonly performed to generate geometrically consistent solid models for dental implant FEA studies, as recommended in recent methodological reviews on finite element methods in implant dentistry [18].

Computer-aided design (CAD) procedures were carried out using SolidWorks^®^ 2020 SP 5.0 (Dassault Systèmes, Vélizy-Villacoublay, France) and SpaceClaim^®^ 2020 R2 (ANSYS Inc., Canonsburg, PA, USA). Surface patching and healing techniques were applied to eliminate geometric discontinuities prior to meshing, as recommended for implant-supported prosthetic simulations [19].

The atrophic maxilla was modeled according to the Cawood and Howell classification, corresponding to a clinical scenario suitable for rehabilitation under the All-on-Four treatment concept [20]. Anatomical structures, including the maxillary sinus and nasal cavity, were preserved to respect surgical and anatomical limitations for implant placement.

Cortical bone thickness was standardized at 1.5 mm based on anatomical averages reported in previous biomechanical studies [21]. The reconstructed geometry also respected typical anatomical dimensions reported for atrophic maxillae, including reduced alveolar ridge height and width characteristic of Cawood and Howell class V–VI conditions. These dimensional features are consistent with previously published morphometric analyses of edentulous maxillae used in implant-supported rehabilitation studies [22].

### 2.2. Implant and Prosthetic Components

**Figure 1:**
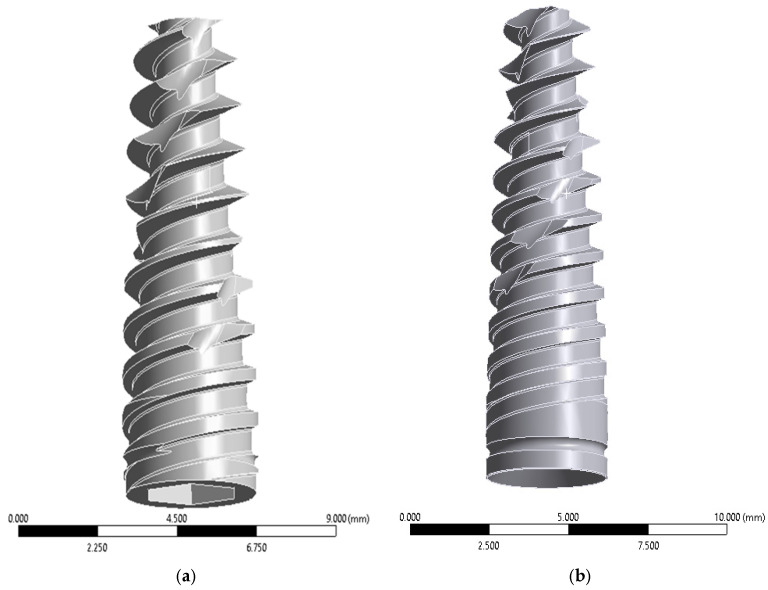
(**a**) An anterior implant measuring 3.75 mm × 11 mm in length; (**b**) a posterior implant measuring 3.75 mm × 13 mm length. Source: own elaboration.

Implant and prosthetic component simplifications (e.g., cylindrical fixtures without explicit thread geometry and rigid bonded contacts) are standard approaches in finite element analysis to reduce computational cost while preserving global stress distribution patterns [23]. Based on this rationale, four implants were modeled according to the All-on-Four protocol (Figure 1).

**Figure 2:**
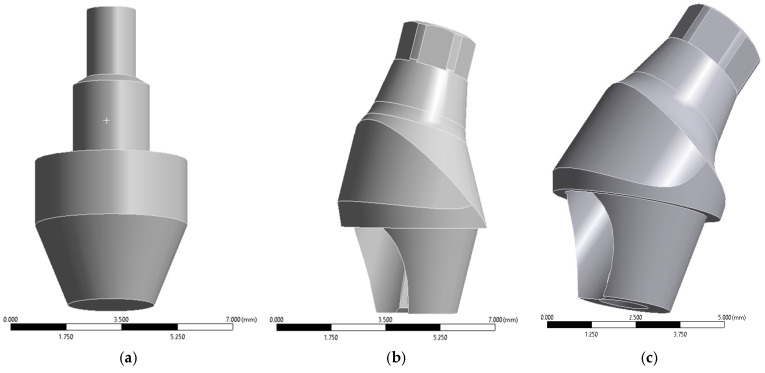
(**a**) Straight (0°); (**b**) 17° angled; (**c**) 30° angled. Source: own elaboration.

Implant geometries were reconstructed in SolidWorks^®^ using nominal commercial dimensions and integrated into the maxillary bone through Boolean subtraction operations. Although thread geometry plays an important role in local stress concentration at the cortical bone interface, previous FEA studies have shown that simplified cylindrical implants preserve global stress distribution trends when comparing implant angulation or prosthetic configurations. Therefore, the present study focused on comparative biomechanical patterns rather than absolute peak stress magnitudes [13]. Mini abutments were modeled in three configurations (Figure 2).

### 2.3. Model Configurations

Generating multiple finite element configurations by varying implant angulation and loading protocols is a common biomechanical comparison strategy in All-on-Four studies [24]. Accordingly, eight finite element models were generated by combining four posterior implant inclinations (0°, 15°, 30°, and 45°) with two loading protocols.

These abutments were assigned according to posterior implant inclinations of 0°, 15°, 30°, and 45°, depending on the experimental configuration, to maintain prosthetic alignment. A maxillary arch-shaped titanium bar was designed with standardized dimensions of 8 mm thickness and 10 mm height. The hybrid prosthesis extended occlusally to the first molar region. All prosthetic components were assumed to be perfectly connected using bonded contact conditions, simulating rigid screw fixation and eliminating micromovement between components.

### 2.4. Material Properties and Contact Conditions

Although human bone exhibits anisotropic and heterogeneous mechanical behavior, modeling bone as a homogeneous isotropic material is a widely adopted assumption in dental implant finite element studies [18,25]. This simplification allows direct comparison with previously published models and significantly reduces computational complexity while preserving the global stress distribution patterns around implants [18,26]. Previous biomechanical investigations have shown that isotropic approximations produce comparable trends in peri-implant stress distribution when evaluating implant angulation or prosthetic design variables implants [18,27]. This modeling approach has been widely adopted in implant biomechanics studies, particularly when the objective is to compare relative stress trends between implant configurations rather than replicate the exact anisotropic behavior of bone tissue [18]. Therefore, the isotropic assumption was considered appropriate for the comparative purposes of this study.

Young’s modulus and Poisson’s ratio values were assigned based on previously validated biomechanical studies [28,29,30]. Two implant–bone interface conditions were simulated:

Delayed loading: Complete osseointegration assumed (bonded contact).

Immediate loading: Frictional contact with a coefficient of friction (µ = 0.3), simulating incomplete osseointegration [25].

Frictional contact was defined using the augmented Lagrange formulation to allow limited relative sliding while maintaining numerical stability.

To ensure numerical consistency, frictional contact conditions were verified and initialized identically on both sides of the maxillary arch before solving the nonlinear models, preventing unintended asymmetries in boundary or contact definitions.

To ensure biomechanical consistency, the assigned elastic moduli were benchmarked against ranges reported in previous finite element studies evaluating implant-supported full-arch rehabilitations [28,29,30]. The cortical and cancellous bone stress magnitudes obtained during preliminary simulations fell within the ranges commonly reported in similar All-on-Four FEA investigations, supporting the external consistency of the model.

### 2.5. Mesh Generation and Convergence Analysis

Mesh convergence analysis is widely adopted to ensure numerical stability in finite element studies of dental implants [23].

Meshing was performed using 10-node quadratic tetrahedral elements (SOLID187, ANSYS 2020 R1 Academic version), selected due to their suitability for irregular geometries and nonlinear contact conditions.

A systematic mesh refinement procedure was conducted. Maximum von Mises stress and displacement values were monitored during progressive refinement. Convergence was defined as achieved when variation between successive refinements was below 5%.

The final mesh consisted of approximately 324,916 nodes. The average element size ranged from 0.5 mm in peri-implant regions to 1.5 mm in less critical anatomical areas.

Additionally, the final maximum von Mises stress values in cortical bone under a 150 N load were compared with those reported in comparable All-on-Four finite element models available in the literature [13,24]. The observed stress levels were within the same order of magnitude as previously published data, indicating appropriate numerical behavior and providing indirect external validation of the computational model.

### 2.6. Boundary Conditions

Boundary restrictions corresponding to anatomical reference planes are commonly employed to prevent rigid body motion in craniofacial finite element simulations [4].

**Figure 3:**
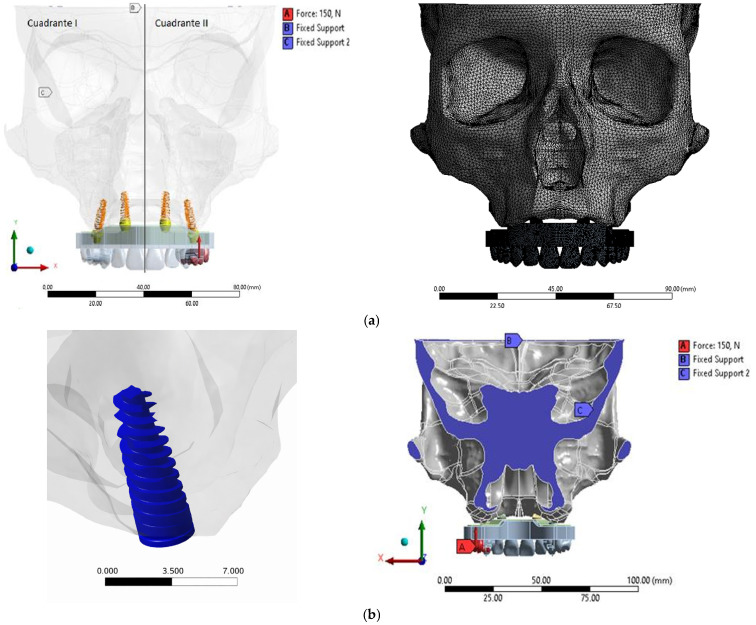
(**a**) Implant locations; (**b**) bonding condition and boundary conditions. Source: own elaboration.

A coronal section passing through the Porion point, aligned with the Frankfort plane, was defined as the primary support region. Two partial displacement constraints were applied (Figure 3).

### 2.7. Loading Conditions

Vertical loads ranging from 100 to 450 N have been reported in finite element studies to simulate posterior masticatory forces [24]. In this study, a static vertical load of 150 N was selected to represent moderate bilateral posterior mastication [31].

The load was distributed over two occlusal contact points located at the first premolar and first molar regions, corresponding to the distal cantilever area of the prosthetic superstructure. Load direction was applied perpendicular to the occlusal plane to standardize mechanical comparison between configurations and isolate the effect of implant angulation.

Vertical loading was selected to isolate the mechanical influence of implant angulation and loading protocol [31,32]. Although oblique and lateral forces occur during mastication, the use of standardized vertical loading is common in comparative FEA studies because it reduces confounding variables when evaluating geometric design parameters.

Systematic analyses of implant FEA models also report that axial loads between 50 and 300 N are the most commonly applied loading conditions in these simulations [33].

For immediate loading simulations, the total applied load was divided into six incremental steps to facilitate nonlinear convergence of the frictional contact model.

The magnitude of the applied load (150 N) and its distribution over posterior occlusal contacts were selected to replicate loading conditions commonly used in implant-supported prosthesis finite element analyses [24]. Preliminary stress outputs were compared with published datasets reporting cortical bone stresses under vertical loads ranging from 100 to 200 N, confirming that the present model produces physiologically plausible mechanical responses.

### 2.8. Outcome Measures

The primary quantitative outcome variable was the maximum von Mises stress in peri-implant cortical bone, as commonly reported in finite element analyses of dental implants [34]. Stress distribution in other components, including cancellous bone, implants, abutments, and bar, was qualitatively evaluated using contour maps, following standard procedures in biomechanical studies [35,36]. Colorimetric contour maps generated in ANSYS^®^ Workbench 20.0.4. were used to identify stress concentration patterns and compare biomechanical performance between configurations, with warm colors indicating regions of higher von Mises stress and cool colors indicating lower stress regions [37].

## 3. Results

### 3.1. Material Properties

All solid bodies were modeled as homogeneous, linearly elastic, and isotropic, consistent with the assumptions described in the methodology. The Young’s modulus and Poisson’s ratio assigned to each material are summarized in Table 1.

### 3.2. Mesh Convergence and Numerical Stability

Mesh independence was verified through a convergence analysis based on the maximum von Mises equivalent stress in peri-implant cortical bone, in accordance with the primary outcome defined in the methodology.

The mesh was progressively refined until variations in stress values between successive refinements were below 5%, in accordance with the convergence criterion defined in the methodology.

All final simulations were performed using approximately 324,916 nodes and 10-node quadratic tetrahedral elements (SOLID187), which provide improved numerical accuracy in complex anatomical geometries such as the maxilla and implant structures.

**Figure 4:**
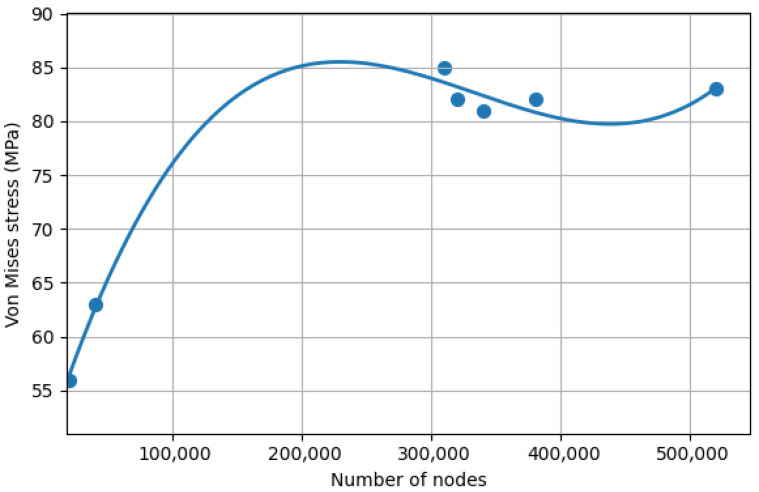
Mesh convergence analysis showing the relationship between the number of nodes and the resulting von Mises stress. Experimental points are presented together with a smooth approximation curve to illustrate the convergence behavior. Source: own elaboration.

Immediate loading simulations required nonlinear contact computation and therefore presented higher computational cost, with solving times between 90 and 100 h per model. Output files reached approximately 30 GB per simulation (Figure 4).

### 3.3. Stress Distribution Analysis

Finite element simulations were performed using ANSYS^®^ Workbench 20.0.4.

Eight three-dimensional models were analyzed, combining four posterior implant inclinations (0°, 15°, 30°, and 45°) with two loading protocols (immediate and delayed).

Table 2 details the number of nodes per component for each implant inclination. Immediate and delayed loading models shared identical mesh configurations to ensure direct comparability of stress results.

Von Mises equivalent stress was evaluated in the peri-implant cortical bone and compared across implant positions and loading conditions.

### 3.4. Immediate Loading

Under immediate loading conditions (frictional interface, µ = 0.3), the highest stress concentration was observed in the posterior left implant at 0° inclination (82.36 MPa). A noticeable asymmetry between the left and right posterior implants was observed in some configurations, particularly at 0° inclination.

A progressive decrease in posterior stress was observed with increasing implant inclination; 0° = 82.36 MPa; 15° = 67.62 MPa; 30° = 45.95 MPa; and 45° = 33.63 MPa.

This represents a stress reduction of approximately 59% between 0° and 45° inclination. Anterior implants exhibited substantially lower stress values across all configurations (range: 2.57–10.77 MPa) (Table 3).

### 3.5. Delayed Loading

Under delayed loading conditions (bonded interface), the highest stress was also recorded at 0° inclination in the posterior left implant (70.5 MPa).

A similar decreasing trend was observed with increasing angulation; 0° = 70.5 MPa; 15° = 66.68 MPa; 30° = 44.46 MPa; 45° = 27.94 MPa. Compared with immediate loading, posterior stress values were generally lower under delayed loading, particularly at 0° and 45° (Table 4).

### 3.6. Comparative Analysis Between Immediate and Delayed Loading

A direct comparison between immediate and delayed loading conditions was performed to quantify differences in peri-implant cortical bone stress.

In general, higher stress concentrations were observed under immediate loading conditions at lower implant inclinations, particularly at 0° and 15° in posterior implants.

The largest absolute difference was recorded at 0° inclination in the posterior left implant, where immediate loading produced 11.86 MPa higher stress than delayed loading.

However, this trend was not uniform across all implant positions. In specific configurations, particularly at higher angulations (30° and 45°) in anterior implants, delayed loading showed slightly higher stress values compared to immediate loading.

These findings indicate that the biomechanical influence of loading protocol depends on both implant inclination and implant position within the arch (Table 5).

## 4. Discussion

Through finite element simulations, this study confirmed the significant influence of posterior implant inclination on peri-implant stress under both immediate and delayed loading. Implants at 0° exhibited the highest stress concentrations, while 45° implants demonstrated the lowest. Delayed loading consistently reduced stress levels compared to immediate loading, aligning with prior reports highlighting the biomechanical advantages of osseointegrated interfaces [13,38,39].

A marked asymmetry in stress distribution between the left and right posterior implants was observed in the present model, particularly under immediate loading conditions. Although the occlusal load was applied bilaterally, several biomechanical factors may explain this difference. First, small geometric variations inherent to the reconstructed maxillary model can influence local stiffness and load transfer paths in finite element simulations [40,41]. Second, the presence of a distal cantilever and the rigid cross-arch framework may produce non-uniform load redistribution, concentrating stresses preferentially on one side of the prosthetic structure. Third, the frictional contact condition used to simulate immediate loading (µ = 0.3) introduces nonlinear behavior at the bone–implant interface, which may lead to asymmetric load transfer even under nominally symmetric loading conditions [42,43]. Similar asymmetric stress patterns have been reported in previous nonlinear FEA studies of implant-supported full-arch prostheses.

Although finite element analysis provides a controlled environment to study biomechanical behavior, its findings should be interpreted in the context of clinical evidence. Clinical studies evaluating the All-on-Four concept have reported high survival rates and favorable functional outcomes when posterior implants are tilted between 30° and 45° [8,9]. For example, long-term clinical follow-up studies have demonstrated survival rates exceeding 95% for tilted implants used in full-arch rehabilitations [8,44]. The stress reduction observed in the present computational model with increasing implant inclination is consistent with these clinical observations, which attribute improved biomechanical behavior to increased anterior–posterior spread and reduced cantilever length [5].

Immediate loading is increasingly recognized as a viable approach for patients achieving primary implant stability, and provided micromotion at the bone–implant interface remains within thresholds conducive to osseointegration (typically <100 µm) [45]. In the present study, simulated implant displacement under immediate loading remained below 4 µm across all inclinations, suggesting favorable conditions for osseointegration. This result reinforces the clinical success of the All-on-Four concept, where immediate splinting via a cross-arch bar and the use of mini-tapered abutments help distribute stress evenly, minimizing the risk of peri-implant bone resorption or prosthetic failure [1,38].

Consistent with previous finite element studies, 30° and 45° posterior tilts produced more favorable stress distribution than 0° and 15° models. While tilting implants increases stress locally due to oblique loading, shortening cantilever length compensates for this effect, reducing overall peri-implant stress [43,46]. The findings highlight that implant angulation alone does not determine stress magnitude; prosthetic design and cantilever length are critical modifiers [47].

In the present study, the posterior left implant consistently exhibited the highest stress values under both immediate and delayed loading conditions, particularly at 0° inclination. This finding is directly reflected in Table 3 and Table 4, where the posterior left implant reached peak stresses of 82.36 MPa under immediate loading and 70.5 MPa under delayed loading. These nuances underscore the complex interplay between implant inclination, cantilever geometry, and loading protocol [48].

This apparent inconsistency may be explained by the redistribution of stress in bonded interfaces. Although osseointegration generally improves load transfer efficiency, it may also transmit forces more directly to the surrounding cortical bone depending on implant position and prosthetic framework rigidity [49]. In contrast, frictional contacts allow limited micro-movement at the implant–bone interface, which may dissipate part of the applied load through sliding effects [50]. Therefore, localized increases in stress under bonded conditions do not necessarily contradict biomechanical theory but rather reflect differences in load transfer mechanisms within the implant–bone interface [51].

In addition, anterior implants occasionally exhibited higher stress values at larger angulations under bonded conditions. This effect may be related to load redistribution through the cross-arch prosthetic framework. As posterior implants become more inclined and absorb a greater proportion of occlusal load, secondary load transfer through the rigid bar structure may increase stress in anterior implants. Similar redistribution patterns have been described in biomechanical analyses of full-arch implant-supported prostheses [43,47].

Despite providing valuable biomechanical insights, this study has several limitations inherent to finite element simulations. First, the finite element models assumed homogeneous, isotropic, and linearly elastic bone properties, which do not fully capture the anisotropic and viscoelastic behavior of human bone [18,34]. In addition, the peak cortical bone stresses observed in some configurations approached the upper range reported for cortical bone in finite element simulations [4,52]. Because the present model assumes isotropic material properties, the absolute stress magnitudes should be interpreted cautiously and primarily as comparative biomechanical trends rather than exact physiological stress values [18,53]. Second, implant threads and certain microstructural geometries were simplified and contacts were idealized to reduce computational cost, which may underestimate localized stress concentrations at the bone–implant interface [18,27,51]. Third, only static vertical loading was simulated, whereas mastication in vivo involves dynamic and multidirectional forces [16,27,34]. Finally, although the computational model allows a controlled comparison of implant inclinations and loading protocols, clinical outcomes may vary due to patient-specific factors such as bone quality, anatomical variability, occlusal patterns, and prosthetic design [18,43]. Therefore, the results should be interpreted as biomechanical predictions rather than direct clinical outcomes, and future in vitro, in vivo, and clinical studies are required to validate these findings before direct translation to patient care.

In summary, tilting posterior implants to 30–45° combined with controlled immediate loading offers a biomechanically favorable approach, reducing stress in peri-implant bone and improving full-arch maxillary rehabilitation outcomes, while recognizing that patient-specific factors and biological variability must guide clinical decision-making.

## 5. Conclusions

This finite element study demonstrated that posterior implant angulation significantly influences peri-implant stress distribution in maxillary All-on-Four rehabilitations. Implants placed at 0° inclination produced the highest stress concentrations in peri-implant cortical bone, whereas increasing posterior angulation to 30° and 45° progressively reduced stress levels. Immediate loading generally resulted in higher peri-implant stress compared with delayed loading, particularly at lower implant inclinations. However, differences between loading protocols became less pronounced at higher angulations.

From a biomechanical perspective, tilting posterior implants between 30° and 45° appears to improve load distribution and reduce stress concentrations in peri-implant bone, supporting the mechanical rationale of the All-on-Four concept.

Future studies should incorporate anisotropic bone modeling, dynamic loading conditions, and patient-specific anatomical geometries to further refine biomechanical predictions. Additionally, long-term clinical studies are needed to validate the mechanical advantages suggested by computational simulations.

## Figures and Tables

**Table 1:** Young’s modulus and Poisson’s ratio.

Material	Young’s Modulus/GPa	Poisson’s Ratio	Reference
Cortical bone	13.7	0.30	[16]
Cancellous bone	1.85	0.30	[16]
Titanium alloy	113	0.35	[16]
Co-Cr alloy	218–220	0.30	[17]
Acrylic resin	~3	0.30	[17]

Source: own elaboration.

**Table 2:** Number of finite element nodes per anatomical and prosthetic component according to posterior implant inclination.

Inclination	Skull	Prostheses	Bar	A Mini Straight Abutment	Anterior Implant 3.75 mm × 11 mm	Mini Angled Abutment	Posterior Implant 3.75 mm × 13 mm
0°	78,106	84,399	54,779	16,019	12,732	15,991	17,021
15°	78,158	84,318	55,642	16,019	12,759	11,307	16,991
30°	78,118	84,351	55,474	15,991	12,785	10,508	17,024
45°	78,215	84,479	55,008	15,991	12,769	10,527	17,069

Source: own elaboration.

**Table 3:** Low immediate loading.

Inclination	Anterior Right	Posterior Right	Anterior Left	Posterior Left
0°	10.77	4.66	6.56	82.36
15°	6.03	7.81	2.57	67.62
30°	3.04	5.2	4.13	45.95
45°	4.38	2.84	6.43	33.63

Source: own elaboration.

**Table 4:** Low late loading.

Inclination	Anterior Right	Posterior Right	Anterior Left	Posterior Left
0°	5.28	10.9	6.22	70.5
15°	3.18	3.86	3.03	66.68
30°	5.61	2.82	6.19	44.46
45°	5.25	3.32	7.93	27.94

Source: own elaboration.

**Table 5:** Difference in the effect of delayed vs. immediate loading on von Mises stress distribution (MPa) in the peri-implant bone.

Inclination	Anterior Right	Posterior Right	Anterior Left	Posterior Left
0°	5.49 *	6.24 Δ	0.34 *	11.86 *
15°	2.85 *	3.95 *	0.46 Δ	0.94 *
30°	2.57 Δ	2.38 *	2.06 Δ	1.49 *
45°	0.87 Δ	0.48 Δ	1.5 Δ	5.69 *

*: Difference in the immediate loading was higher, Δ: difference when the late loading was higher. Source: own elaboration.

## Data Availability

The datasets presented in this article are not readily available because they are part of a larger ongoing research project and contain extensive computational simulation data that require specialized processing and storage. Requests to access the datasets should be directed to the corresponding author.
